# The short-chain fatty acid butyrate exerts a specific effect on VE-cadherin phosphorylation and alters the integrity of aortic endothelial cells

**DOI:** 10.3389/fcell.2023.1076250

**Published:** 2023-02-08

**Authors:** Jiangang Guo, Inka Terhorst, Paul Stammer, Abdulhakim Ibrahim, Alexander Oberhuber, Thorsten Eierhoff

**Affiliations:** Department for Vascular and Endovascular Surgery, University Hospital Münster, Münster, Germany

**Keywords:** aortic endothelial cell, butyrate, free fatty acid receptor (FFAR), VE-cadherin, phosphorylation, permeability, short-chain fatty acid (SCFA), c-src

## Abstract

Short-chain fatty acids (SCFAs) like butyrate (BUT) largely influence vascular integrity and are closely associated with the onset and progression of cardiovascular diseases. However, their impact on vascular endothelial cadherin (VEC), a major vascular adhesion and signaling molecule, is largely unknown. Here, we explored the effect of the SCFA BUT on the phosphorylation of specific tyrosine residues of VEC (Y731, Y685, and Y658), which are reported to be critical for VEC regulation and vascular integrity. Moreover, we shed light on the signaling pathway engaged by BUT to affect the phosphorylation of VEC. Thereby, we used phospho-specific antibodies to evaluate the phosphorylation of VEC in response to the SCFA sodium butyrate in human aortic endothelial cells (HAOECs) and performed dextran assays to analyze the permeability of the EC monolayer. The role of c-Src and SCFA receptors FFAR2 and FFAR3 in the induction of VEC phosphorylation was analyzed using inhibitors and antagonists for c-Src family kinases and FFAR2/3, respectively, as well as by RNAi-mediated knockdown. Localization of VEC in response to BUT was assessed by fluorescence microscopy. BUT treatment of HAOEC resulted in the specific phosphorylation of Y731 at VEC with minor effects on Y685 and Y658. Thereby, BUT engages FFAR3, FFAR2, and c-Src kinase to induce phosphorylation of VEC. VEC phosphorylation correlated with enhanced endothelial permeability and c-Src-dependent remodeling of junctional VEC. Our data suggest that BUT, an SCFA and gut microbiota-derived metabolite, impacts vascular integrity by targeting VEC phosphorylation with potential impact on the pathophysiology and therapy of vascular diseases.

## 1 Introduction

A balanced opening and closure of endothelial junctions maintain the vascular integrity that allows a certain plasticity in response to angiogenic and inflammatory stimuli. Dysregulation of endothelial integrity leading to enhanced permeability is often a precondition to arterial disease (Mundi et al., 2018). Vascular endothelial cadherin (VEC), an approx. 140 kDa transmembrane protein, is an essential regulator of the endothelial integrity by its adhesive and signaling properties (Harris and Nelson, 2010). It is widely accepted that phosphorylation of tyrosines in the cytoplasmic domain of VEC can destabilize adherence junctions and increase the permeability of endothelial cell (EC) monolayers. VEC is phosphorylated in response to angiogenic and inflammatory factors such as VEGF and TNF-α, respectively, which results in the enhanced phosphorylation of tyrosine-731 and -658 of VEC and increased permeability of the EC monolayer *in vitro* and *in vivo* (Monaghan-Benson and Burridge, 2009; Wessel et al., 2014). The phosphorylation of tyrosine-731 and -658 VEC prevents p-120 catenin and β-catenin from binding to VEC (Potter et al., 2005) and can lead to a reduction in the plasma membrane retention of the VEC complex (Lampugnani and Dejana, 2007; Dejana et al., 2008).

Circulating metabolites such as short-chain fatty acids (SCFAs) exert different effects on endothelial integrity and permeability (Amedei and Morbidelli, 2019). SCFAs are fatty acids of up to six carbon atoms of length, which are produced mainly through fermentation of dietary fiber by the gut microbiota, with butyrate (BUT), propionate, and acetate as the most abundant SCFAs. Several studies have shown a significant correlation of gut microbiota-derived metabolites such as SCFAs with the onset and progression of cardiovascular diseases (Wang et al., 2011; Karoor et al., 2021). A low microbial BUT-producing potential is linked with heart failure and coronary artery disease (Troseid et al., 2020). At the cellular level, SCFAs have been demonstrated to interfere with endothelial activation by decreasing the production of inflammatory cytokines IL-6 and IL-8 and the expression of adhesion molecules such as VCAM-1 in response to LPS- and TNF-alpha stimulation (Li et al., 2018b; Li et al., 2018c), as well as the expression of ICAM-1 and E-selectin (Miller et al., 2005). These processes seem to be mechanistically linked to the binding of SCFAs to the G-protein-coupled receptors (GPCR) FFAR3 and FFAR2 (formerly GPCR41 and GPCR43, respectively) and to the inhibition of histone deacetylases (HDACs) (Li et al., 2018a). Furthermore, SCFAs amerliorate Ang-II-induced endothelial dysfunction by interfering with NADPH-derived ROS production (Robles-Vera et al., 2020). Moreover, SCFAs, especially BUT, not only strengthen the intestinal epithelial barrier (Peng et al., 2009) but also improve the junctional integrity of venous EC (Miyoshi et al., 2008).

While VEC phosphorylation and EC permeability in response to angiogenic and inflammatory stimulation have been intensively studied, little is known about the direct impact of SCFAs on VEC phosphorylation. Several studies suggest a role for BUT in stabilizing the endothelial barrier function involving VEC. However, it is not known whether BUT might directly regulate VEC and which kinases or phosphatases are involved. Moreover, many studies in vascular biology have been conducted using venous (umbilical) EC, whereas the function and regulation of VEC in EC of (large) arterial vessels like the aorta is poorly understood.

Therefore, we investigated the effect of BUT on the phosphorylation of VEC and the integrity of primary human aortic endothelial cells (HAOEC). We demonstrate that BUT induces a remodeling of VEC, which correlates with a measurable impact on aortic endothelial permeability. We found that increased monolayer permeability is correlated with enhanced VEC phosphorylation at tyrosine-731, which depended on functional SCFA receptors FFAR2/3. Furthermore, we demonstrate that c-Src kinase mediates BUT-induced VEC phosphorylation.

## 2 Materials and methods

### 2.1 Cell culture

Primary human aortic endothelial cells (HAOECs) were purchased from PromoCell (Heidelberg, Germany, c-12271, LOT#4082102.16). Cells were cultured at 37°C and 5% CO_2_ in a humidified incubator in endothelial cell growth medium MV (PromoCell, Heidelberg, Germany, C-22020) and endothelial cell supplement mix (PromoCell, Heidelberg, Germany, c-39225). For all experiments, the cells were used at passage 4 to 6. Cells were grown to 100% confluence and then stimulated with Ang-II (Sigma Merck, A9525), sodium butyrate (Millipore, 567430), PP2 (Sigma, 529573), GLPG0974 (Sigma, SML2443), and ß-HB (β-hydroxybutyric acid) (Sigma, 166898). Sodium butyrate was resolved in pH-buffered endothelial cell basal medium (PromoCell, c-22220) without supplements (pH ±SD at 37°C and 5% CO_2_ measured in triplicate by a pH meter: pH_0.1mM BUT_ = 7.36 ± 0.04; pH_1mM BUT_ = 7.38 ± 0.02; and pH_5mM BUT_ = 7.35 ± 0.05). Control cells were treated only by basal medium (pH = 7.36 ± 0.03) without a vehicle.

### 2.2 Human biospecimens

Tissue of human thoracic aorta was obtained during surgery and fixed in 4% PFA immediately after resection for immunohistochemistry (see Immunohistochemistry). Patient consent for the collection and use of the samples was obtained in advance, based on a positive vote by the ethics committee.

### 2.3 Antibodies

We used the following commercially available antibodies for western blotting and immunofluorescence microscopy: monoclonal mouse anti-p-Tyr (PY99, Santa Cruz, sc-7020), polyclonal rabbit anti-phospho-VEC (against phospho-Tyr731, Invitrogen 44-1145G) (Luo et al., 2017), polyclonal rabbit anti-phospho-VEC (against phospho-Tyr685, Abcam, ab119785) (Liu et al., 2021), polyclonal rabbit anti-phospho-VEC (against phospho-Tyr658, Invitrogen, 44-1144G) (Hahn et al., 2015), monoclonal mouse anti-human VEC (clone F-8, Santa Cruz, sc-9989), monoclonal mouse anti-human VEC (BV6, Millipore, MABT134), monoclonal mouse anti-human SRC (Invitrogen, AHO1152), monoclonal rabbit anti-human phospho-SRC (against phospho-Tyr 416 (D49G4), Cell Signaling, 6943), monoclonal rabbit anti-human VEC (E6N7A, Cell Signaling, 93467S), and monoclonal mouse anti-human ß-actin (Cell Signaling, 3700). The secondary antibodies include goat anti-rabbit IgG HRP-linked antibody (Cell Signaling, 7074), horse anti-mouse IgG HRP-linked antibody (Cell Signaling, 7076), polyclonal goat anti-mouse IgG (H + L) cross-adsorbed Alexa Fluor 488 (Invitrogen, A11001), goat anti-mouse IgG (H + L) Cy3 (Jackson ImmunoResearch, 111-165-062), and donkey anti-rabbit IgG (H + L) Alexa Fluor 488 (Invitrogen, A21206).

### 2.4 Western blotting

Cells were lysed in RIPA lysis buffer (150 mM NaCl (AppliChem, 141659.1211), 50 mM Tris-HCl (pH 8.0) (Roth, 4855,1), 1% Triton X-100 (AppliChem, A4975), 0.5% w/v sodium deoxycholate (AppliChem, A1531.0100), 0.1% w/v SDS (AppliChem, A1112.0500) supplemented with protease inhibitor cocktail (Sigma, P8340), and sodium orthovanadate (Sigma, 6508)). After incubating on ice for 1 h, the cell lysates were cleared by centrifugation at 20,817 rpm for 10 min at 4°C. The concentration of total proteins was measured using the Pierce BCA Protein Assay Kit (Thermo Fisher Scientific, 23225). The lysates were mixed with the sample buffer (250 mM Tris-HCl, pH 6.8) (Roth, 4855.2), 30% v/v glycerin (Fisher Scientific, 10021083), 10% w/v SDS, 0.5 M 1,4-dithiothreitol (DTT, Roth, 6908.1), and 0.5% w/v Bromphenolblau (Serva, 15375.02)) and heated at 100°C for 10 min. 10 µg of cell lysates were loaded on 10% polyacrylamide gel (SDS-PAGE) (resolving gel (2.5 mL running buffer (1.5 M Tris-HCL, pH 9.0), 4% v/v TEMED (Roth, 2367.1), 0.4% w/v SDS), 4.02 mL Millipore water, 3.38 mL Rotiphorese^®^ Gel 30 (Roth, 3029.2), 45 µL 10% v/v ammonium persulfate (Roth, 9592.5), stacking gel (4.44 mL stacking buffer (140 mM Tris-HCl (PH 6.8), 0.1% v/v TEMED, 0.1% w/v SDS), and 650 µL Rotiphorese^®^ Gel 30, 100 µL 10% v/v ammonium persulfate) and electro-transferred into a 0.45-µm nitrocellulose membrane (Roth, 9201.1). The membranes were then incubated with 10% low fat milk (MILSANI, Germany) in TBST (TBS containing 0.05% v/v Tween-20 (AppliChem, A4974) at room temperature for 1 h. Primary antibodies (phospho-Tyr731(1 μL/mL), phospho-Tyr658 (2 μL/mL), phospho-Tyr658 (2 μL/mL), VEC (F-8) (0.2 μg/ml), ß-actin (0.5 μL/mL), and SRC (1 μg/ml) were diluted in 10% w/v low fat milk in TBST and incubated at 4°C overnight. After washing three times for 10 min in TBST, the membranes were incubated with secondary antibodies for 45 min at room temperature, followed by detection by chemiluminescence (Clarity ECL; BioRad, 1705061) and imaging on a ChemiDoc Imaging System (BioRad). Protein abundance was quantified by densitometry (Image Lab software version 6.0.1) and subsequently normalized to a loading control. Phosphorylated VEC was detected by an HRP-linked secondary antibody (0.5 μL/mL), while the total VEC was detected by the cross-adsorbed Alexa Fluor 488 secondary antibody (4 μg/ml).

### 2.5 Endothelial permeability assay

HAOECs were seeded on top of a polyester membrane transwell insert on 12-well-plates (12 mm Transwell^®^ with 0.4-μm Pore Polyester Membrane Insert, Corning, #3460/TC-Inserts, 0.4 mM Pore, Sarstedt, 83.3931.041 for knockdown experiments) at a density of 0,8 × 10^5^ cells/chamber and cultured for 72 h. After discarding the medium, the cells in the upper chamber were incubated with different concentrations of BUT as indicated. Subsequently, the medium in the apical chamber was replaced by endothelial cell basal medium containing 0.05 mg/ml of 10 kDa dextran (Invitrogen, D22910) followed by incubation for 30 min at 37°C in a CO_2_ incubator, after which the fluorescence (F) intensity in the upper and lower chambers was determined with a 96-well plate reader (Synergy HTX Multifunction Detector, BioTek, United States: excitation 485/20 and emission 528/20). All independent experiments were performed in triplicate. Permeability was presented as F_basal_/F_apical_ (F_b_/F_a_). After VEC was knocked down by specific siRNA, permeability was measured as described previously and experiments were performed in duplicate.

### 2.6 Immunofluorescence microscopy

In brief, HAOECs were seeded into a 24-well plate (1 × 10^5^ cells/well) on glass coverslips for 48 h. Confluent monolayers of HAOECs were treated with 1 mM of BUT for 1 h. Afterward, HAOECs were fixed with 4% v/v paraformaldehyde in PBS (Roth, 0335.3) for 1 h and incubated with 50 mM NH_4_CL (Roth, K298.1) for 45 min at room temperature. HAOECs were incubated with the anti-VEC antibody (E6N7A and BV6, respectively) diluted 1:100 in non-permeabilizing blocking solution (5% w/v Albumin Fraction V (Roth, T844.1) in PBS) or anti-phospho-tyrosine antibody (PY99) diluted 1:100 in permeabilizing blocking solution (containing 0.3% v/v Triton^®^ X-100, AppliChem, A4975,0500) for 60 min at room temperature. Coverslips were washed three times with PBS. HAOECs were incubated with a secondary antibody of Alexa Flour 488 anti-mouse IgG (H + L) or Alexa Fluor 488 donkey anti-rabbit IgG (H + L) and goat anti-rabbit IgG (H + L) Cy3 diluted 1:300 for 45 min, washed three times with PBS, and then incubated for 5 min at room temperature with a mounting medium (Dako, S3023). Images were acquired using a fluorescence microscope (Nikon, ECLIPSE Ti2) and NIS-Elements AR 5.02.03 and NIS-Elements Viewer 5.21. For quantification of junction phenotypes, we used the program junction mapper (Brezovjakova et al., 2019). After the map was created, all cell boundaries of representative cells were measured by Junction Mapper. The thresholds for the VEC signal were set to zero signals in the cytoplasm. Only completely sharp boundaries with continuous contact with adjacent cells were included into the analysis. Data were shown from at least three independent experiments.

### 2.7 Immunohistochemistry

HAOECs and tissue of human thoracic aorta were fixed in 4% PFA/PBS and embedded in paraffin. Before paraffin embedding, 2.5 × 10^6^ HAOEC in 50 µL PBS were mixed in an Eppendorf tube with 50 µL of 10 mg/mL agarose (Sigma, A9539-25G) in PBS at 42°C. Agarose-embedded HAOECs were placed in a histological cassette for further paraffinization. Sections of 4 µm were cut with a rotary microtome (Leica, Germany). Histological sections were dried overnight at 37°C. After deparaffinization and dehydration, the sections were pretreated with target retrieval solution (Dako, S1699, pH 6.1) for 40 min in a steamer. Intrinsic peroxidase was blocked with 3% H2O2/PBS. FFAR3 primary antibodies (GPR41 Rabbit anti-human, Polyclonal, Invitrogen, and PA599629) and FFAR2 (GPR43 Rabbit Polyclonal Antibody, Invitrogen, and PA5-100944) were diluted in antibody diluent (Dako REAL, S2022). Sections were incubated with a primary antibody overnight at 4°C in a humid chamber. Sections were then incubated with a polymer-enhancer and HRP-polymer from the SuperVision 2 HRP Kit (DCS, PD000POL) for 1 h, respectively. Diaminobenzidine tetrahydrochloride hydrate (DAB, Sigma, D5637) was used as a color substrate and nucleus staining was performed with hematoxylin. Finally, the sections were mounted using the Eukitt^®^ mounting medium (Kindler). Histological sections were analyzed using an upright microscope (Nikon, ECLIPSE Ci) and images were acquired using NIS-Elements F 4.60.00 and NIS-Elements Viewer 5.21 software. For quantification of FFAR3 expression, images were converted into 8-bit TIFs and cell borders of cells in the focal plane were outlined manually, defining the ROI. Mean signal intensities of ROIs were extracted using ImageJ.

### 2.8 Cellular viability and proliferation

The MTT assay was used to determine the cell viability after adding BUT using the MTT Assay Kit by Promega (G4000, including Solubilization Solution/Stop Mix G401A and Dye Solution G402A). HAOECs were seeded into a 96-well plate at a density of 1 × 10^4^ cells per well in 100 μL of the complete medium and grown to confluence. Each group contained three replicates. The cells were treated with various doses of BUT (0.1 mM, 1 mM, and 5 mM) for 1 h at 37°C and 5% CO_2_ in a humidified incubator. Afterward, 15 μL dye solution was added to each well and the cells were incubated for 4 h at 37°C in a CO_2_ incubator. Crystals were solubilized by adding 100 µL Solubilization Solution/Stop Mix for 1 h. The content of each well was mixed to obtain a uniformly colored solution by using a multichannel pipette. Absorbance at 570 nm wavelength was measured using a plate reader (BioTek).

### 2.9 Transfection of siRNA

HAOECs were seeded into a 12-well plate (1.2 × 10^5^ cells/well) and the cell density reached 70%–80% confluence before transfection with siRNA. The following reagents were used for transfection: control siRNA-A (Santa Cruz, sc-37007), siRNA transfection medium (Santa Cruz, sc-36868), siRNA transfection reagent (Santa Cruz, sc-29528), siRNA dilution buffer (sc-29517), VE-cadherin siRNA (h) (Santa Cruz, sc-36814), GPR41 siRNA(h) (Santa Cruz, sc-97148), and siRNA c-Src (h) (Santa Cruz, sc-29228). The cells were transfected according to the manufacturer’s instructions (Protocol “siRNA Mediated Inhibition of Gene Expression” by Santa Cruz). In brief, control siRNA-A and target siRNA were diluted in 66 μL and 330 µL siRNA dilution buffer, respectively. 4 µL of each siRNA duplex solution and transfection reagent, respectively, were added into 100 µL of the transfection medium, gently mixed, and incubated for 30 min. 792 µL of the transfection medium was added to each tube containing the siRNA and reagent, mixed gently, and incubated with cells. After culturing at 37°C and 5% CO_2_ for 5.5 h, the transfection medium was replaced by endothelial cell growth medium, and experiments were performed between 12 and 24 h after transfection.

### 2.10 Quantitative RT-PCR (RT-qPCR)

The total RNA of 80×10^5^ HAOECs was extracted using the RNeasy Mini-Kit (50) (QIAGEN, 74104) according to the manufacturer’s instructions. 100 ng of total RNA was reverse-transcribed using the iScript™ Advanced cDNA Synthesis Kit (Bio-Rad, 1725037) at 46°C for 20 min and 95°C for 1 min in a thermal cycler (Mastercycler nexus gradient, Eppendorf). 100 ng of cDNA was used for qPCR performed with SsoAdvanced Universal SYBR^®^ Green Supermix (Bio-Rad, 1725270) according to the manufacturer’s instructions with 1 µL of primers for FFAR2 (Bio-Rad, qHsaCED0044139), FFAR3 (Bio-Rad, qHsaCED0037214), and Glyceraldehyde 3-phosphate dehydrogenase (GAPDH, Bio-Rad, qHsaCED0038674). Samples were measured using the CFX96 system (Bio-Rad) and analyzed using BioRad Analysis Software (Maestro). Relative mRNA levels of FFAR3 were normalized to GAPDH. PCR products were subjected to agarose gel electrophoresis (75V, 60 min) on a 1% agarose gel stained with GelStain-Red dye (Roth, 0984.1).

### 2.11 Statistical analysis

Statistical analysis was carried out with GraphPad Prism 9 software (GraphPad Software, San Diego, CA, United States). All data are presented as the mean ± SEM. Data were analyzed for normality and equal variance by the Shapiro–Wilk test. To compare between multiple experimental groups, non-parametric data were analyzed with the Kruskal–Wallis test, while parametric data were analyzed with one-way analysis of variance (ANOVA), followed by Tukey’s *post hoc* analysis. An unpaired *t*-test (Welch´s *t*-test) was used to compare two independent samples (Figure 3C). Statistical significance was considered for *p* ≤ 0.05 (*), *p* ≤ 0.005 (**), *p* ≤ 0.001 (***), and *p* ≤ 0.0001 (****).

## 3 Results

### 3.1 BUT enhances the permeability of aortic EC monolayers depending on VEC

**FIGURE 1:**
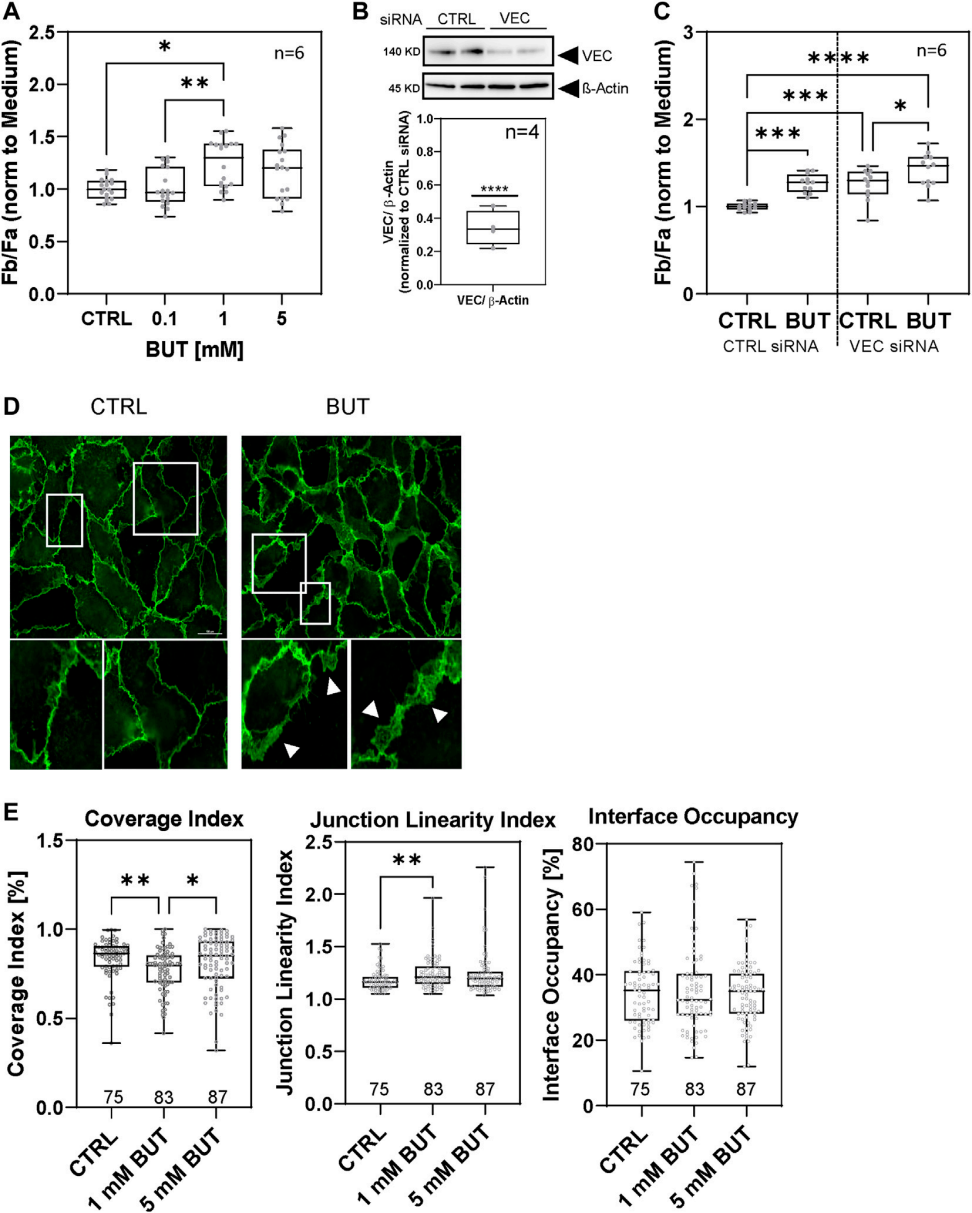
BUT increases permeability in a VEC-dependent manner in HAOECs. **(A)** Permeability of the HAOEC monolayer was checked by stimulating HAOECs with different concentrations of BUT for 1 h. Fb/Fa: ratio of fluorescence intensity in the basal chamber (Fb) and fluorescence intensity apical chamber (Fa). **(B)** KD of VEC expression in HAOEC with a decrease of 65.99% was confirmed by western blotting. **(C)** Permeability of the HAOEC monolayer as described in **(A)** in the control (CTRL) and VEC KD cells. Cells were treated with 1 mM BUT for 1 h. **(A**, **C)** Data represent mean values of n = 6 experiments each measured in biological duplicate **(C)** or triplicate **(A)**. Statistical significance was considered for *p* ≤ 0.05 (*), *p* ≤ 0.005 (**), *p* ≤ 0.001 (***), and *p* ≤ 0.0001 (****). **(D)** Immunofluorescence microscopy images of non-permeabilized HAOEC stained for junctional VEC (antibody clone E6N7A) after 1 mM BUT or medium-only (CTRL) treatment for 1 h. Images 1–4 represent zoomed-in areas indicated by white rectangles with arrowheads pointing to specific remodeled junctional regions. Images show representative data of n = 3 independent experiments. Scale bar (100 μm). **(E)** Quantification of the junctional VEC pattern for VEC coverage, interface linearity, and VEC interface occupancy using the program Junction Mapper (see Materials and Methods). Numbers of analyzed junctions of n = 3 experiments are given underneath the bars.

Since previous studies have demonstrated the impact of BUT on the endothelial barrier function, we first tested the capacity of BUT to alter the permeability of the HAOEC monolayer. Therefore, HAOECs were exposed for 1 h to different concentrations of BUT from 0.1 to 5 mM in serum-free growth medium (Figure 1). Of note, BUT induced a measurable increase in EC monolayer permeability at 1 mM characterized by a significant increase in FITC-Dextran diffusion (10 kDa) from the upper to the lower compartment of the filter inserts. At 1 mM and 5 mM BUT, diffusion was increased by about 1.24 ± 0.05 fold and 1.18 ± 0.06 fold, respectively, whereas 0.1 mM BUT moderately decreased EC permeability, compared to the medium (Figure 1A). A knockdown (KD) of VEC expression by siRNA (Figure 1B) significantly increased EC permeability *per se* in contrast to cells transfected with non-targeting siRNA (Figure 1C), indicating that the integrity of the HAOEC monolayer relies on VEC expression. Of note, under the VEC knockdown condition, BUT was still able to increase the permeability, but the magnitude given by the difference between BUT-treated and –untreated cells (0.1663) is lower than that of cells transfected with non-targeting siRNA (0.2656) (Figure 1C). These findings indicate that BUT exerts its effect on aortic endothelial permeability *via* VEC probably in addition to other pathways.

Since VEC mediates BUT-induced effects on HAOEC permeability, we next checked the localization of VEC at the cell junctions by fluorescence microscopy to determine whether junctional VEC was modified in response to BUT. We observed an altered VEC localization in HAOECs in response to BUT-treated cells, which resembled interrupted, plaque-like patterns for VEC, suggesting a remodeling of junctional VEC by BUT (Figure 1D).

We further characterized the patterns of VEC by analyzing the images obtained by immune fluorescence microscopy using Junction Mapper software (Brezovjakova et al., 2019). Junction Mapper identifies cellular junction semi-automated and analyzes the length, intensity, and distribution of junctional protein staining. To compare morphology in BUT-treated cells to the control, we used indices not affected by the intensity to prevent influence of variance of staining. The Coverage Index measures the level of stained fragments along the cellular junction, the Junction Linearity Index measures the deviation from a straight-line junction, and the Interface Occupancy measures the share of stained area in a fixed area around the junction.

The analysis confirmed a significantly higher degree of fractionated, junctional VEC (Coverage Index) in BUT-treated cells (1 mM) than in control cells (Figure 1E). Moreover, the linearity of VEC-distribution at cellular junctions (Junction Linearity Index) of BUT-treated cells (1 mM) is decreased compared to control cells (Figure 1E).

Interestingly, increased permeability and junction remodeling occurred not due to potential toxic effects of BUT since under all experimental conditions we did not measure relevant effects on cell viability and toxicity (Supplementary Figure S1). Because these results hint on a direct impact of BUT on VEC, we subsequently examined whether distinct phosphorylation sites of VEC, which are critical for its regulation, are affected by BUT treatment.

### 3.2 BUT increases the specific tyrosine phosphorylation of VEC

**FIGURE 2:**
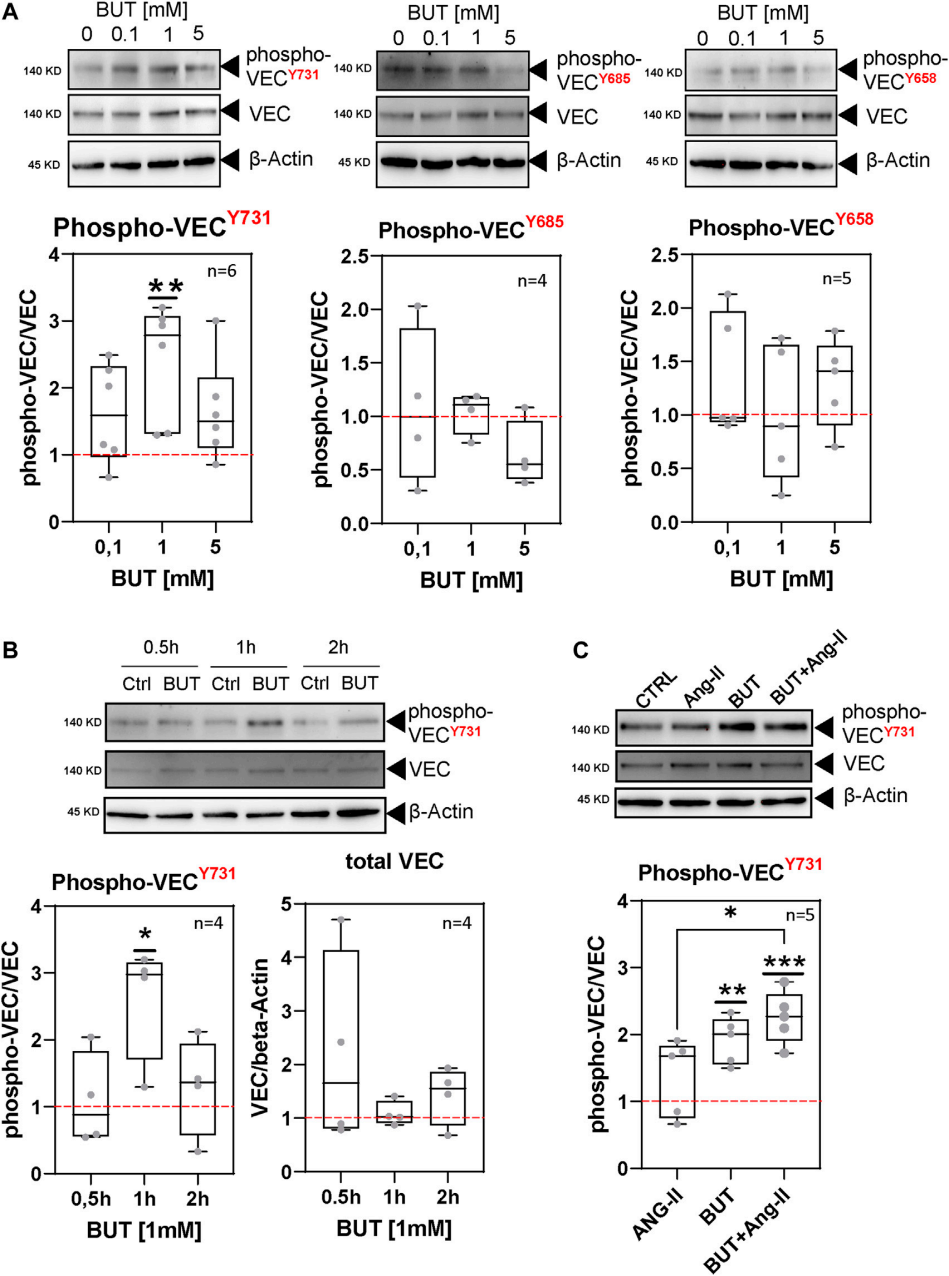
BUT induces specific tyrosine phosphorylation of VEC. **(A)** Phosphorylation of VEC at tyrosine-731, -685, and -658 (phospho-VECY731, Y685, and Y658) in response to various concentrations of BUT analyzed by western blotting. HAOECs were treated for 1 h with indicated concentrations of BUT. Phospho-VEC signals where normalized to the total VEC. Relative phospho-VEC signals were normalized to the level in control cells (dashed red line). β-Actin served as a loading control. **(B)** Kinetic of BUT-induced phospho-VECY731 level analyzed by western blotting. Data display densiometric quantification of relative phospho-VEC and VEC signals normalized to the level in control cells (dashed red line) of n ≥ 4 experiments. For better illustration, the identical dataset for 1 h treatment at 1 mM from panel **(A)** (phospho-VECY731) is shown. **(C)** Phospho-VECY731 level after 1 h treatment of HAOEC with BUT (1 mM) in the presence or absence of Ang-II (100 ng/mL). Data display densitometric quantification of relative phospho-signals as described in **(B)**.

We analyzed the phosphorylation status of three tyrosine residues of VEC, which have been reported to regulate endothelial permeability: tyrosine-731, -685, and -658. Therefore, we incubated HAOECs under the same conditions described for the permeability experiments (Figure 1A) with 0.1 mM, 1 mM, and 5 mM BUT and analyzed the tyrosine-specific phosphorylation of VEC in cell lysates by western blotting followed by densitometry quantification. Thereby, we used validated antibodies which detect specific phosphorylation of VEC at tyrosine-731, -685, and -658 (Hahn et al., 2015; Luo et al., 2017; Liu et al., 2021). We found that BUT affects the phosphorylation levels of all target tyrosines to varying degrees. The largest effects could be observed for tyrosine-731, where BUT led to elevated phosphorylation compared to medium-only treated cells (Figure 2A). VEC phosphorylation for tyrosine-731 peaked at 1 mM of BUT (Figure 2A, far left panel). Following the analysis of the time course of phosphorylation revealed that tyrosine-731 was transiently phosphorylated in response to BUT (1 mM) which peaked at 1 h of treatment by 2.40 ± 0.35 fold, whereas the phospho-level at 0.5 h and 2 h was just moderately increased compared to the baseline level (red dashed line, Figure 2B). The phospho-VEC-levels of tyrosine-685 and -658 did not significantly change during the observation period compared to medium-only treated cells (Supplementary Figure S2). This suggests that BUT exerts a site-specific effect on VEC. Interestingly, phosphorylation of VEC did not result in the decreased amount of full-length VEC at approx. 140 kDa, which would indicate significant degradation (Figure 2B, lower right panel).

Ang-II is an important angiogenic molecule with a broad impact on vascular (patho-) physiology. Among others, Ang-II influences EC permeability and VEC phosphorylation (Wu et al., 2016). Therefore, we analyzed whether BUT may interfere with or enhance the effects of Ang-II regarding VEC phosphorylation. Indeed, we observed an elevated level of phospho-tyrosine-731 in Ang-II (100 ng/ml)-treated cells (Figure 2C) in line with previous reports (Jeong et al., 2019). Interestingly, BUT was able to increase this level when co-incubated with Ang-II (Figure 2C), suggesting a synergistic rather than an interfering effect of BUT on Ang-II function with respect to VEC phosphorylation.

In summary, BUT stimulated a measurable increase in tyrosin-731 phosphorylation at VEC in a time- and dose-dependent manner, which correlated with an increased permeability of the HAOEC monolayer (Figure 1).

### 3.3 FFAR2 and FFAR3 are engaged by BUT to increase the phosphorylation of VEC

**FIGURE 3:**
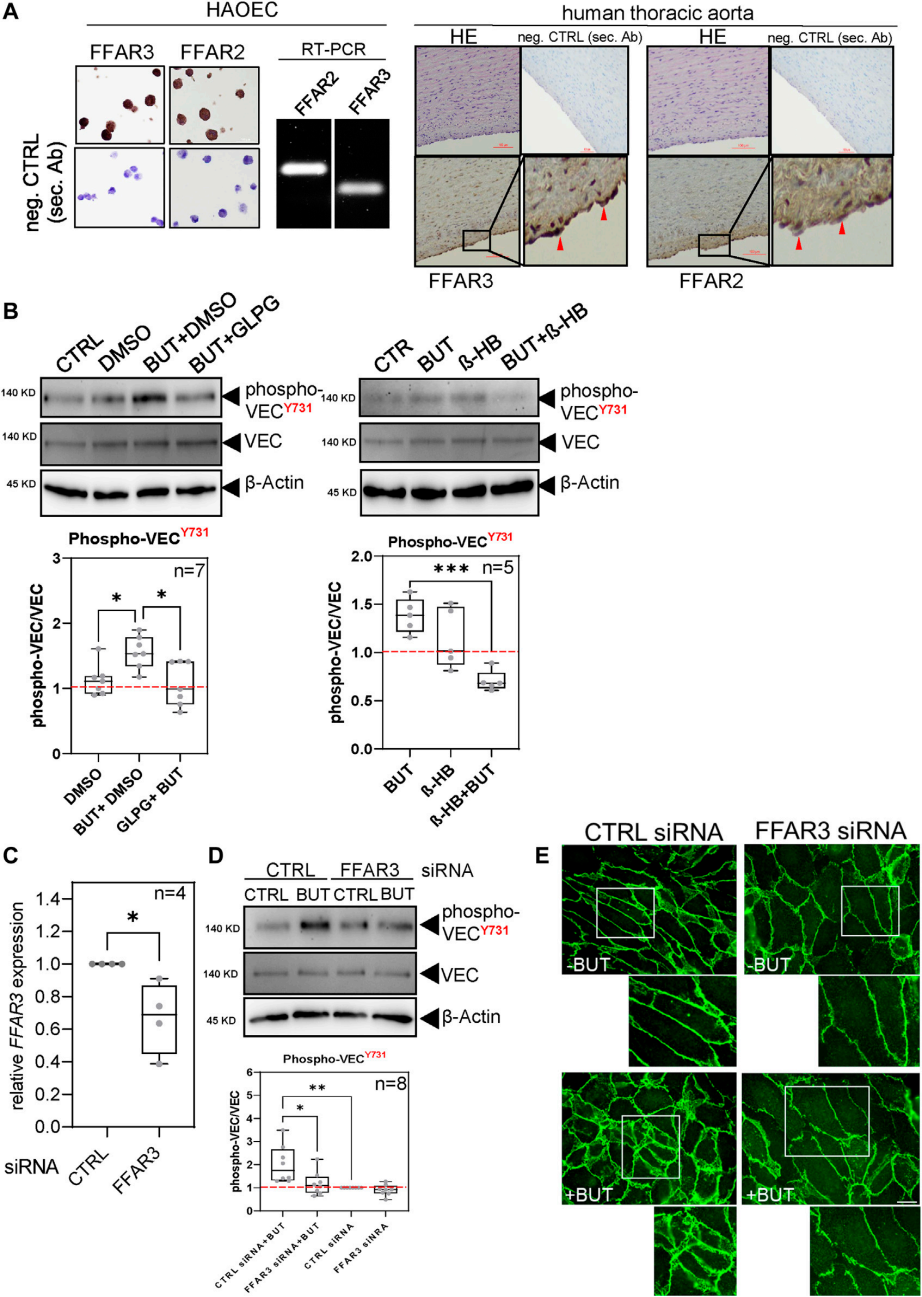
Perturbation of FFAR2/3 expression and function diminishes BUT-induced VEC phosphorylation. **(A)** Detection of FFAR2/3 expression by RT-PCR and immunohistochemistry (IHC). IHC staining was performed on fixed and agarose-embedded HAOEC (left panel) and of FFPE samples of human thoracic aorta (right panel). Images below represent zoomed-in areas indicated by black rectangles with red arrowheads pointing to specific receptor staining. Scale bar (100 μm). **(B)** BUT-induced VEC phosphorylation analyzed as shown in Figure 2 upon pre-incubation overnight with 0.1 µM of FFAR3 antagonist GLPG0974 (left panel) and co-incubation with FFAR2 antagonist ß-HB (0.1mM, right panel). For the GLPG0974 approach, cells were treated either with a vehicle alone (DMSO) or in combination with BUT and GLPG0974. Data represent n ≥ 5 experiments. **(C)** Validation of FFAR3 KD by RT-qPCR. Relative expression of *FFAR3* normalized to *GAPDH* between CTRL (=1) and FFAR3 siRNA-transfected HAOEC of n = 3 independent transfection experiments. **(D)** BUT-induced VEC phosphorylation analyzed as shown in Figure 2 in the presence and absence of FFAR3 KD of n = 6 experiments. In FFAR3 KD cells, Y731 phosphorylation in response to butyrate treatment was decreased by 40.91% compared to scrambled siRNA. **(E)** Immunofluorescence microscopy images of non-permeabilized HAOEC stained for junctional VEC (antibody clone BV6) after 1 mM BUT or medium-only (-BUT) treatment for 1 h. Cells were transfected with control or FFAR3 siRNA under the same conditions used in **(C**, **D)**. Small images represent enlarged areas of the sections marked by white rectangles. Images show representative data of n = 3 independent experiments. Scale bar (100 μm).

FFAR2 and FFAR3 bind SCFAs including BUT and trigger intracellular signaling pathways. Therefore, we experimentally addressed the question of whether FFAR2 and FFAR3 are involved in the BUT-induced VEC phosphorylation in HAOECs. Detection of FFAR2 and FFAR3 by immunohistochemistry and RT-PCR using commercially available antibodies and primers confirmed the expression of these receptors in HAOECs (Figure 3A left panel) and in the intima of human aorta (Figure 3A right panel, red arrowheads). We initially perturbed the receptor function using an antagonist of FFAR2 (GLPG0974) and FFAR3 (β-HB) (Figure 3B). In the presence of GLPG, phospho-tyrosine-731 significantly dropped in HAOEC, whereas in the absence of the antagonist, BUT increased the phospho-tyrosine-731 level (Figure 3B, left panel). When BUT was co-incubated with β-HB, the resulting phospho-tyrosine-731 level was decreased to the basal level as observed for GLPG (Figure 3B, right panel). Since BUT has higher affinity for FFAR3 than for FFAR2 (Brown et al., 2003), we subsequently performed a siRNA-mediated knockdown of FFAR3 and tested if BUT was still able to elevate the phospho-tyrosine-731 level of VEC as observed previously (Figure 2A). Even when FFAR3 expression was only moderately knocked down (Figure 3C, Supplementary Figure S3), BUT failed to efficiently induce the phosphorylation of tyrosine-731, in contrast to cells properly expressing FFAR3 (Figure 3D). This raises the question if BUT-induced junctional remodeling of VEC (Figure 1D) also relies on FFAR3 expression. Indeed, we observed that BUT was less efficiently able to remodel junctional VEC in cells treated with FFAR3 siRNA. Under such a condition, VEC localization principally resembled those in BUT-unstimulated HAOECs (Figure 3E), indicating a role for FFAR3 not only in BUT-induced phosphorylation but also for junctional remodeling of VEC.

These findings suggest that FFAR2/3 is involved in the signaling pathway engaged by BUT that finally leads to the phosphorylation and junctional remodeling of VEC.

### 3.4 c-Src kinases mediate VEC phosphorylation induced by BUT

**FIGURE 4:**
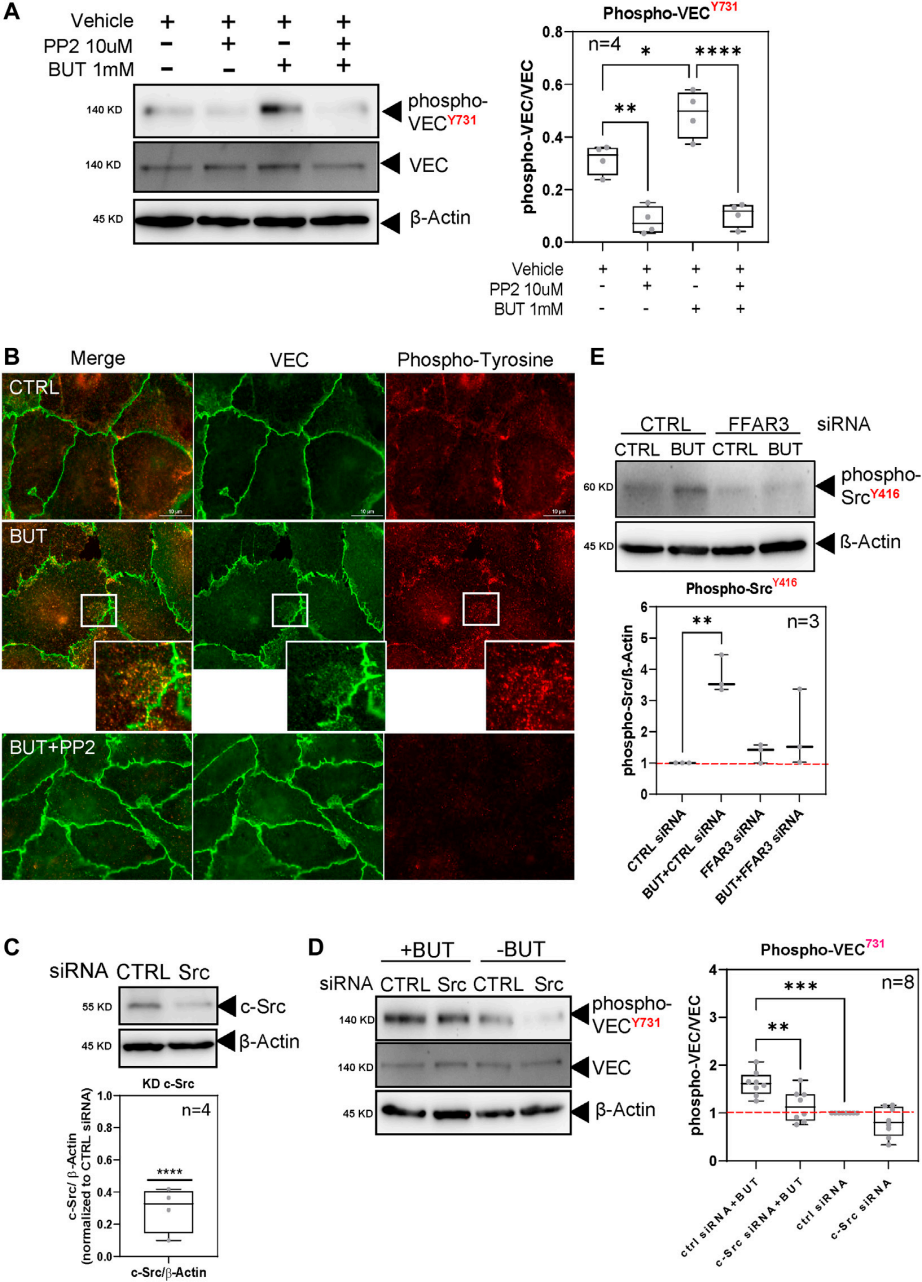
BUT engages Src kinases for specific tyrosine phosphorylation at VEC. **(A)** HAOECs were treated for 15 min before and during BUT incubation with PP2 (10 μM) or vehicle (DMSO). Subsequently, phospho-VECY731 levels were analyzed by western blotting. Phospho-signals of n = 4 experiments were quantified by densitometry. **(B)** Immune fluorescent microscopy of HAOECs treated with the vehicle (DMSO) or PP2 and BUT (5 mM) for 1 h and subsequently stained for VEC and general phospho-tyrosine residues. Scale bar (10 μm). **(C)** Validation of Src KD by RNAi. HAOECs were transfected with Src-specific siRNA or control-siRNA. Cells were lysed and analyzed for Src expression 12 h later by western blotting. **(D)** Stimulation of Src KD and control cells with BUT (1 mM) for 1 h and analysis of phosho-VECY731 expression as shown in Figure 2. Quantification corresponds to n = 6 experiments. **(E)** Phospho-SrcY416 western blotting of BUT-treated or -untreated cells, transfected with Ctrl or FFAR3 siRNA. Data display densiometric quantification of relative phospho-Src signals normalized to the level in BUT-untreated Ctrl siRNA-transfected cells (dashed red line) of n = 3 experiments.

c-Src kinase has been reported to phosphorylate specific tyrosine residues at VEC to regulate the stability of VEC complexes and endothelial permeability. We initially detected that the basal phosphorylation of VEC at tyrosine-731 was significantly lowered upon the application of the broadband inhibitor of Src family kinases PP2 (Figure 4A). This finding confirmed that c-Src kinases are mainly involved in the phosphorylation of VEC also in aortic EC. When PP2 was applied in combination with BUT, phospho-tyrosine-731 remained at the level observed for PP2-only treated HAOECs (Figure 4A), whereas BUT alone was still able to induce VEC phosphorylation (Figure 4A) to a similar extent as observed previously (Figure 2). This suggests that BUT likely activates Src family kinases to phosphorylate VEC. Interestingly, we found by immune-fluorescence microscopy that an altered junctional VEC pattern in response to BUT (Figure 4B middle panel; Figure 1D) was partially reversed and resembled VEC junctions in the control approach, when BUT was co-incubated with PP2 (Figure 4B, lower panel). Moreover, BUT increased the general phospho-tyrosine level in a c-Src-dependent manner, which was partially co-localized with junctional VEC compared to PP2-untreated cells, (Figure 4B, zoom-in panel). Finally, we tested by specific KD of c-Src expression (Figure 4C) if c-Src indeed mediates BUT-induced VEC phosphorylation. In HAOECs not stimulated with BUT, the c-Src KD condition *per se* led to a similar decrease in the basal phosphorylation of VEC at tyrosine-731 as observed for PP2 (Figure 4D). When HAOECs were stimulated with BUT upon KD of c-Src, BUT was still able to increase the phosphorylation of VEC at tyrosine-731 but to a significantly lower level than in cells transfected with non-targeting siRNA (Figure 4D). The findings demonstrate that c-Src is, indeed, engaged by BUT to phosphorylate VEC at tyrosine 731. Since our data suggest that Src kinase activation is required to phosphorylate VEC, we next analyzed if Src becomes activated downstream of FFAR3. Therefore, we probed cell lysates of FFAR3 KD and control cells (Figure 3D) for Src activation by detecting phosho-Src^Y416^ (Adam et al., 2010). Thereby, we found an increased level of phospho-Src^Y416^ in response to BUT treatment (Figure 4E, CTRL siRNA), whereas the phosphorylation of Src was less pronounced by BUT in FFAR3 KD cells (Figure 4E, FFAR3 siRNA). This indicates that FFAR3 is required for Src activation.

In summary, our results suggest that BUT may influence specific VEC phosphorylation by activating a signaling cascade in aortic EC triggered by the binding of BUT to FFAR2/3 which results in the activation of c-Src as an effector kinase to phosphorylate and remodel junctional VEC. These processes presumably lead to the observed increased endothelial permeability of aortic EC monolayer under BUT treatment.

## 4 Discussion

It is widely accepted that VEC is a critical factor for vascular homeostasis involved in the regulation of EC permeability. VEC is regulated by tyrosine phosphorylation (Orsenigo et al., 2012; Wessel et al., 2014). Studies have shown that angiogenic factors such as VEGF or inflammatory molecules and cytokines such as LPS and TNF-α specifically influence the phosphorylation of VEC. Instead, nothing is known about the influence of circulating SCFAs on the specific regulation of VEC, although they demonstrate various effects on cell physiology. Here, we show that the SCFA BUT solely and specifically influences the phosphorylation of VEC in aortic EC. Depending on which tyrosine is phosphorylated or dephosphorylated at VEC, cell junctions are stabilized or destabilized. The induction of the phosphorylation of tyrosine-731 and -658 at VEC by c-Src and Pyk2 in conjunction with the engagement of ICAM-1 was demonstrated to be required for efficient transendothelial migration of leucocytes (Allingham et al., 2007). We found that BUT enhances EC permeability and stimulates the phosphorylation of tyrosine-731 at VEC, whereas the phosphorylation of tyrosine-685 was decreased by higher BUT concentrations (5 mM). Although it is tempting to speculate here whether BUT may facilitate diapedesis by enhancing the phosphorylation of tyrosine-731, a recent study demonstrated that dephosphorylation of tyrosine-731 by SHP-2 promotes leukocyte diapedesis upon ICAM-1 mediated attachment, whereas the phosphorylation of tyrosine-685 selectively increases endothelial permeability (Wessel et al., 2014). This clearly shows that the regulation of junctional integrity *in vivo* is more complex and the generalizations of the *in vitro* effects of BUT on the regulation of VEC in vascular EC are, therefore, limited. Further studies evaluating the effects of BUT (and other SCFAs) on specific regulatory sites of VEC *in vivo* are urgently needed in view of possible (patho-) physiological correlations and endothelial integrity. Our data suggest that BUT engages c-Src for the phosphorylation and junctional remodeling of VEC. These findings are in line with several studies demonstrating that c-Src phosphorylates specific tyrosine at VEC, although there is a debate on which tyrosines c-Src preferentially phosphorylate and if this correlates with decreased endothelial barrier function (Adam et al., 2010), whereas some studies report tyrosine-658 and -731 to be the main targets for c-Src; others report that tyrosine-685 is exclusively phosphorylated by c-Src (Wallez et al., 2007). Beyond a direct effect on c-Src, BUT may also indirectly affect c-Src activity, for example, by inhibition of Csk which would lead to enhanced activity as suggested by studies using dominant negative Csk which demonstrate the induction of VEC phosphorylation at tyrosine-731, -658, and -685 (Adam et al., 2010). However, since our data indicate the specific phosphorylation of tyrosine 731 in response to BUT, the mode of action of BUT on the c-Src-VEC axis remains to be investigated.

One physical parameter among others, which probably is most influential on VEC phosphorylation, is shear force. Interestingly, among the phospho-sites at VEC we inspected, tyrosine-658 seems to be a site which is phosphorylated in response to laminar flow, which peaked at 3.5 dyne cm^−2^ (Orsenigo et al., 2012). In our static-cultured ECs, BUT failed to exert any detectable effect on this site, which may be related to the lack of shear force as a prerequisite for its regulation. Therefore, future experiments in our laboratory will focus on this aspect to explore (BUT-regulated) phospho-sites at VEC, which are sensitive to shear force to match physiological conditions.

SCFAs such as BUT bind to and activate G-protein-coupled receptors FFAR2 and FFAR3 with different selectivities (Brown et al., 2003; Bindels et al., 2013). FFAR2 and FFAR3 are expressed in adipose tissue and immune cells (Brown et al., 2003), as well as in hepatocytes and the intestinal epithelium (Chambers et al., 2015; Lopez-Mendez et al., 2021). Their expression in vascular EC is still a matter of debate and, consequently, little is known if and how FFAR2/3 functions in vascular EC. Studies demonstrated the expression in vascular EC (Li et al., 2018a) with undetectable to low expression for FFAR3 in aorta (Regard et al., 2008; Pluznick et al., 2013). We detected signals of FFAR2 and FFAR3 in aortic EC and in the intima of human aorta, thereby supporting the findings of other studies (Natarajan et al., 2016; Li et al., 2018a). Activated FFAR2 and FFAR3 trigger downstream signaling which leads to the inhibition of adenylate cyclase (AC) and decreased levels of cAMP and increased intracellular calcium. Moreover, activated FFAR2 inhibits NFκB *via* β-arrestin-2, thereby lowering pro-inflammatory IL-1β and IL-6 levels (Meijer et al., 2010). In human monocytes and macrophages, FFAR2/3 can build heteromers with distinct signaling, which, for example, lack the ability to decrease cAMP production (Ang et al., 2018). In our study, we found that application of compounds known to antagonize FFAR2/3 impacts BUT-induced VEC phosphorylation, suggesting that FFAR2/3 activation might be involved in BUT-induced VEC phosphorylation. To our knowledge, it was yet unknown that FFAR2/3 signaling also influences VEC phosphorylation. We found that Src is activated by BUT in an FFAR3-dependent manner (Figure 4E). How could FFAR3 regulate VEC phosphorylation *via* Src? An increased cAMP level activates protein kinase A and subsequently, Csk to inactivate Src (Abrahamsen et al., 2003). A possible mechanism for a BUT-induced signaling cascade, therefore, would be that the cellular cAMP level is lowered by the inhibition of AC in response to FFAR3 activity, which *vice versa* inactivates Csk, leading to the activation of Src for VEC phosphorylation.

However, we did not test for activation of FFAR2/3 nor analyze the type of FFAR2/3 dimerization. These questions remain unanswered and the subject of our further investigation to shed light on the still poorly understood role of these receptors in vascular biology in general and in the context of VEC regulation in particular.

BUT has been demonstrated to increase the endothelial and epithelial barrier function by inhibiting the effects induced by TNF-a and LPS (Parada Venegas et al., 2019; Karoor et al., 2021). We observed that BUT transiently increased the permeability of the HAOEC monolayer within 1 h of stimulation, indicating at least temporarily, suspended barrier function. Remarkably, this increase in permeability occurs without detectable degradation of full-length VEC and without measurable impairment of cell viability in our experiments. Instead, we found BUT to induce VEC remodeling, resembling plaque-like and interrupted junctional VEC (Figure 1D; Figure 4B). EC junction remodeling associates with (myosin-mediated) lamellipodia formation (Doggett and Breslin, 2011) and phosphorylation of VEC (Caolo et al., 2018). This raises the questions of whether BUT by phosphorylation of VEC rather stimulates the formation of junction-associated intermittent lamellipodia (JAIL), which have been reported to produce very similar VEC dynamics and are important for monolayer integrity (Abu Taha et al., 2014; Cao and Schnittler, 2019). In the clinical context, increased endothelial permeability could be detrimental, for example, in the process of atherosclerosis. Paradoxically, BUT has been described to exert atheroprotective effects on cardiovascular diseases (reviewed by Amiri et al., 2022). However, elevated EC permeability by BUT as observed in our study may represent increased activity and remodeling at cell junctions leading to the formation of new junctional complexes during tissue regeneration and wound healing processes. In this regard, our data would still support a beneficial role for butyrate and only seem to contradict the effects of BUT reported in other studies.

Phosphorylation of VEC at tyrosine-731 and -658 prevents binding of β-catenin and p120-catenin to VEC and inhibits barrier function (Potter et al., 2005). One may speculate that BUT-induced phosphorylation of VEC consequently lowers membrane retention of VEC complexes due to impaired binding of p120-catenin to VEC to allow such dynamics for the remodeling of junctional VEC as observed in our experiments. However, whether BUT stimulates JAIL-mediated VEC dynamics in HAOECs remains to be elucidated, particularly the role of Arp2/3-branched actin, from which JAIL formation may originate (Cao and Schnittler, 2019).

SCFAs like BUT were shown to be closely associated with cardiovascular diseases. The effects of SCFAs and BUT at a systemic and cellular level during aortic pathology are not understood.

Ang-II is a critical factor at the systemic and cellular level for the pathogenesis of vascular diseases in general (Berk et al., 2000) and, in particular, for aortic diseases like aortic dissection and aneurysms (Lagrange et al., 2020; Wu et al., 2021). Experimental work suggests that Ang-II in conjunction with RAGE (receptor for advanced glycation end products) increases phospho-VEC tyrosine-731 and VEC disruption that correlate with the induction of hyperpermeability of HUVECs and murine aortas (Jeong et al., 2019). We could show that Ang-II also increases VEC phosphorylation at tyrosine-731 in aortic EC and, furthermore, that BUT enhances this effect in combination with Ang-II. This raises the question of whether BUT (or other SCFAs/circulating gut microbiota metabolites) may amplify Ang-II function with respect to its hyperpermeability properties, during pathologic vascular processes and prospectively determine the onset and progression of aortic pathologies. This is especially interesting to answer for aortic dissection, as here the intimal (endothelial) layer is ruptured, that is putatively related to VEC disruption and EC hyperpermeability. Assessment of patients regarding blood levels of circulating SCFAs, especially of BUT, may clarify if the onset and progression of aortic diseases associate with the distinct level of specific SCFAs such as BUT.

It was demonstrated that patients with symptomatic atherosclerosis and coronary artery disease lack BUT-producing gut microbiota (Karlsson et al., 2012; Liu et al., 2019), concluding that BUT may have beneficial effects on the onset and progression of cardiovascular diseases. However, little is known about the effects of BUT on the cellular level and in the context of aortic diseases. *In vivo* data support a protective role for BUT in conjunction with its receptor FFAR3 in the development of neointima hyperplasia after injury of femoral artery (Nooromid et al., 2020). Interestingly, we found FFAR3 to be engaged by BUT for VEC phosphorylation, which correlates with remodeling of cell junctions. Given that EC junctions play a critical role in regeneration (Evans et al., 2021), our data could hint on a potential role for BUT during intima regeneration by remodeling of junctional VEC, which is initially triggered by FFAR3-induced VEC phosphorylation.

Taken together, our study provides new insights into the interaction of BUT, a SCFA and microbiota-derived metabolite, with VE-cadherin in aortic endothelial cells. Future research should now clarify potential clinical implications also including other SCFAs.

## Data Availability

The original contributions presented in the study are included in the article/Supplementary Material further inquiries can be directed to the corresponding author.

## References

[B1] Abrahamsen H. Vang T. Taskén K. (2003). Protein kinase A intersects SRC signaling in membrane microdomains. J. Biol. Chem. 278 (19), 17170–17177. 10.1074/jbc.M211426200 12606547

[B2] Abu Taha A. Taha M. Seebach J. Schnittler H. J. (2014). ARP2/3-mediated junction-associated lamellipodia control VE-cadherin-based cell junction dynamics and maintain monolayer integrity. Mol. Biol. Cell 25 (2), 245–256. 10.1091/mbc.E13-07-0404 24227887 PMC3890345

[B3] Adam A. P. Sharenko A. L. Pumiglia K. Vincent P. A. (2010). Src-induced tyrosine phosphorylation of VE-cadherin is not sufficient to decrease barrier function of endothelial monolayers. J. Biol. Chem. 285 (10), 7045–7055. 10.1074/jbc.M109.079277 20048167 PMC2844154

[B4] Allingham M. J. van Buul J. D. Burridge K. (2007). ICAM-1-mediated, Src- and Pyk2-dependent vascular endothelial cadherin tyrosine phosphorylation is required for leukocyte transendothelial migration. J. Immunol. 179 (6), 4053–4064. 10.4049/jimmunol.179.6.4053 17785844

[B5] Amedei A. Morbidelli L. (2019). Circulating metabolites originating from gut microbiota control endothelial cell function. Molecules 24 (21), 3992. 10.3390/molecules24213992 31694161 PMC6864778

[B6] Amiri P. Hosseini S. A. Ghaffari S. Tutunchi H. Ghaffari S. Mosharkesh E. (2022). Role of butyrate, a gut microbiota derived metabolite, in *cardiovascular diseases*: A comprehensive narrative review. Front. Pharmacol. 12, 837509. 10.3389/fphar.2021.837509 35185553 PMC8847574

[B7] Ang Z. Xiong D. Wu M. Ding J. L. (2018). FFAR2-FFAR3 receptor heteromerization modulates short-chain fatty acid sensing. FASEB J. 32 (1), 289–303. 10.1096/fj.201700252RR 28883043 PMC5731126

[B8] Berk B. C. Haendeler J. Sottile J. (2000). Angiotensin II, atherosclerosis, and aortic aneurysms. J. Clin. Invest. 105 (11), 1525–1526. 10.1172/JCI9820 10841510 PMC300860

[B9] Bindels L. B. Dewulf E. M. Delzenne N. M. (2013). GPR43/FFA2: Physiopathological relevance and therapeutic prospects. Trends Pharmacol. Sci. 34 (4), 226–232. 10.1016/j.tips.2013.02.002 23489932

[B10] Brezovjakova H. Tomlinson C. Mohd Naim N. Swiatlowska P. Erasmus J. C. Huveneers S. (2019). Junction Mapper is a novel computer vision tool to decipher cell-cell contact phenotypes. Elife 8, e45413. 10.7554/eLife.45413 31793877 PMC7034980

[B11] Brown A. J. Goldsworthy S. M. Barnes A. A. Eilert M. M. Tcheang L. Daniels D. (2003). The Orphan G protein-coupled receptors GPR41 and GPR43 are activated by propionate and other short chain carboxylic acids. J. Biol. Chem. 278 (13), 11312–11319. 10.1074/jbc.M211609200 12496283

[B12] Cao J. Schnittler H. (2019). Putting VE-cadherin into JAIL for junction remodeling. J. Cell Sci. 132 (1), jcs222893. 10.1242/jcs.222893 30606730

[B13] Caolo V. Peacock H. M. Kasaai B. Swennen G. Gordon E. Claesson-Welsh L. (2018). Shear stress and VE-cadherin. Arterioscler. Thromb. Vasc. Biol. 38 (9), 2174–2183. 10.1161/ATVBAHA.118.310823 29930007

[B14] Chambers E. S. Morrison D. J. Frost G. (2015). Control of appetite and energy intake by SCFA: What are the potential underlying mechanisms? Proc. Nutr. Soc. 74 (3), 328–336. 10.1017/S0029665114001657 25497601

[B15] Dejana E. Orsenigo F. Lampugnani M. G. (2008). The role of adherens junctions and VE-cadherin in the control of vascular permeability. J. Cell Sci. 121 (13), 2115–2122. 10.1242/jcs.017897 18565824

[B16] Doggett T. M. Breslin J. W. (2011). Study of the actin cytoskeleton in live endothelial cells expressing GFP-actin. J. Vis. Exp. 57, 3187. 10.3791/3187 PMC3308586 22126853

[B17] Evans C. E. Iruela-Arispe M. L. Zhao Y. Y. (2021). Mechanisms of endothelial regeneration and vascular repair and their application to regenerative medicine. Am. J. Pathol. 191 (1), 52–65. 10.1016/j.ajpath.2020.10.001 33069720 PMC7560161

[B18] Hahn C. S. Scott D. W. Xu X. Roda M. A. Payne G. A. Wells J. M. (2015). The matrikine N-alpha-PGP couples extracellular matrix fragmentation to endothelial permeability. Sci. Adv. 1 (3), e1500175. 10.1126/sciadv.1500175 26229981 PMC4517288

[B19] Harris E. S. Nelson W. J. (2010). VE-Cadherin: At the front, center, and sides of endothelial cell organization and function. Curr. Opin. Cell Biol. 22 (5), 651–658. 10.1016/j.ceb.2010.07.006 20708398 PMC2948582

[B20] Jeong J. Lee J. Lim J. Cho S. An S. Lee M. (2019). Soluble RAGE attenuates AngII-induced endothelial hyperpermeability by disrupting HMGB1-mediated crosstalk between AT1R and RAGE. Exp. Mol. Med. 51 (9), 1–15. 10.1038/s12276-019-0312-5 PMC6802637 31562296

[B21] Karlsson F. H. Fak F. Nookaew I. Tremaroli V. Fagerberg B. Petranovic D. (2012). Symptomatic atherosclerosis is associated with an altered gut metagenome. Nat. Commun. 3, 1245. 10.1038/ncomms2266 23212374 PMC3538954

[B22] Karoor V. Strassheim D. Sullivan T. Verin A. Umapathy N. S. Dempsey E. C. (2021). The short-chain fatty acid butyrate attenuates pulmonary vascular remodeling and inflammation in hypoxia-induced pulmonary hypertension. Int. J. Mol. Sci. 22 (18), 9916. 10.3390/ijms22189916 34576081 PMC8467617

[B23] Lagrange J. Finger S. Kossmann S. Garlapati V. Ruf W. Wenzel P. (2020). Angiotensin II infusion leads to aortic dissection in LRP8 deficient mice. Int. J. Mol. Sci. 21 (14), 4916. 10.3390/ijms21144916 32664652 PMC7404218

[B24] Lampugnani M. G. Dejana E. (2007). Adherens junctions in endothelial cells regulate vessel maintenance and angiogenesis. Thromb. Res. 120, S1–S6. 10.1016/S0049-3848(07)70124-X 18023702

[B25] Li M. van Esch B. Henricks P. A. J. Folkerts G. Garssen J. (2018a). The anti-inflammatory effects of short chain fatty acids on lipopolysaccharide- or tumor necrosis factor alpha-stimulated endothelial cells via activation of GPR41/43 and inhibition of HDACs. Front. Pharmacol. 9, 533. 10.3389/fphar.2018.00533 29875665 PMC5974203

[B26] Li M. van Esch B. Henricks P. A. J. Garssen J. Folkerts G. (2018b). Time and concentration dependent effects of short chain fatty acids on lipopolysaccharide- or tumor necrosis factor alpha-induced endothelial activation. Front. Pharmacol. 9, 233. 10.3389/fphar.2018.00233 29615908 PMC5867315

[B27] Li M. van Esch B. Wagenaar G. T. M. Garssen J. Folkerts G. Henricks P. A. J. (2018c). Pro- and anti-inflammatory effects of short chain fatty acids on immune and endothelial cells. Eur. J. Pharmacol. 831, 52–59. 10.1016/j.ejphar.2018.05.003 29750914

[B28] Liu H. Chen X. Hu X. Niu H. Tian R. Wang H. (2019). Alterations in the gut microbiome and metabolism with coronary artery disease severity. Microbiome 7 (1), 68. 10.1186/s40168-019-0683-9 31027508 PMC6486680

[B29] Liu M. M. Zhou J. Ji D. Yang J. Huang Y. P. Wang Q. (2021). Diammonium glycyrrhizinate lipid ligand ameliorates lipopolysaccharide-induced acute lung injury by modulating vascular endothelial barrier function. Exp. Ther. Med. 21 (4), 303. 10.3892/etm.2021.9734 33717246 PMC7885082

[B30] Lopez-Mendez I. Mendez-Maldonado K. Manzo-Francisco L. A. Juarez-Hernandez E. Uribe M. Barbero-Becerra V. J. (2021). G protein-coupled receptors: Key molecules in metabolic associated fatty liver disease development. Nutr. Res. 87, 70–79. 10.1016/j.nutres.2020.12.019 33601216

[B45] Luo M. Flood E. C. Almeida D. Yan L. Berlin D. A. Heerdt P. M. (2017). Annexin A2 supports pulmonary microvascular integrity by linking vascular endothelial cadherin and protein tyrosine phosphatases. J. Exp. Med. 214 (9), 2535–2545. 10.1084/jem.20160652 28694388 PMC5584111

[B31] Meijer K. de Vos P. Priebe M. G. (2010). Butyrate and other short-chain fatty acids as modulators of immunity: What relevance for health? Curr. Opin. Clin. Nutr. Metab. Care 13 (6), 715–721. 10.1097/MCO.0b013e32833eebe5 20823773

[B32] Miller S. J. Zaloga G. P. Hoggatt A. M. Labarrere C. Faulk W. P. (2005). Short-chain fatty acids modulate gene expression for vascular endothelial cell adhesion molecules. Nutrition 21 (6), 740–748. 10.1016/j.nut.2004.11.011 15925300

[B33] Miyoshi M. Usami M. Ohata A. (2008). Short-chain fatty acids and trichostatin A alter tight junction permeability in human umbilical vein endothelial cells. Nutrition 24 (11-12), 1189–1198. 10.1016/j.nut.2008.06.012 18723323

[B34] Monaghan-Benson E. Burridge K. (2009). The regulation of vascular endothelial growth factor-induced microvascular permeability requires Rac and reactive oxygen species. J. Biol. Chem. 284 (38), 25602–25611. 10.1074/jbc.M109.009894 19633358 PMC2757962

[B35] Mundi S. Massaro M. Scoditti E. Carluccio M. A. van Hinsbergh V. W. M. Iruela-Arispe M. L. (2018). Endothelial permeability, LDL deposition, and cardiovascular risk factors-a review. Cardiovasc Res. 114 (1), 35–52. 10.1093/cvr/cvx226 29228169 PMC7729208

[B36] Natarajan N. Hori D. Flavahan S. Steppan J. Flavahan N. A. Berkowitz D. E. (2016). Microbial short chain fatty acid metabolites lower blood pressure via endothelial G protein-coupled receptor 41. Physiol. Genomics 48 (11), 826–834. 10.1152/physiolgenomics.00089.2016 27664183 PMC6223570

[B37] Nooromid M. Chen E. B. Xiong L. Shapiro K. Jiang Q. Demsas F. (2020). Microbe-derived butyrate and its receptor, free fatty acid receptor 3, but not free fatty acid receptor 2, mitigate neointimal hyperplasia susceptibility after arterial injury. J. Am. Heart Assoc. 9 (13), e016235. 10.1161/JAHA.120.016235 32580613 PMC7670501

[B38] Orsenigo F. Giampietro C. Ferrari A. Corada M. Galaup A. Sigismund S. (2012). Phosphorylation of VE-cadherin is modulated by haemodynamic forces and contributes to the regulation of vascular permeability *in vivo* . Nat. Commun. 3, 1208. 10.1038/ncomms2199 23169049 PMC3514492

[B39] Parada Venegas D. De la Fuente M. K. Landskron G. Gonzalez M. J. Quera R. Dijkstra G. (2019). Short chain fatty acids (SCFAs)-Mediated gut epithelial and immune regulation and its relevance for inflammatory bowel diseases. Front. Immunol. 10, 277. 10.3389/fimmu.2019.00277 30915065 PMC6421268

[B40] Peng L. Li Z. R. Green R. S. Holzman I. R. Lin J. (2009). Butyrate enhances the intestinal barrier by facilitating tight junction assembly via activation of AMP-activated protein kinase in Caco-2 cell monolayers. J. Nutr. 139 (9), 1619–1625. 10.3945/jn.109.104638 19625695 PMC2728689

[B41] Pluznick J. L. Protzko R. J. Gevorgyan H. Peterlin Z. Sipos A. Han J. (2013). Olfactory receptor responding to gut microbiota-derived signals plays a role in renin secretion and blood pressure regulation. Proc. Natl. Acad. Sci. U. S. A. 110 (11), 4410–4415. 10.1073/pnas.1215927110 23401498 PMC3600440

[B42] Potter M. D. Barbero S. Cheresh D. A. (2005). Tyrosine phosphorylation of VE-cadherin prevents binding of p120-and beta-catenin and maintains the cellular mesenchymal state. J. Biol. Chem. 280 (36), 31906–31912. 10.1074/jbc.M505568200 16027153

[B43] Regard J. B. Sato I. T. Coughlin S. R. (2008). Anatomical profiling of G protein-coupled receptor expression. Cell 135 (3), 561–571. 10.1016/j.cell.2008.08.040 18984166 PMC2590943

[B44] Robles-Vera I. Toral M. de la Visitacion N. Aguilera-Sanchez N. Redondo J. M. Duarte J. (2020). Protective effects of short-chain fatty acids on endothelial dysfunction induced by angiotensin II. Front. Physiol. 11, 277. 10.3389/fphys.2020.00277 32372967 PMC7176911

[B46] Troseid M. Andersen G. O. Broch K. Hov J. R. (2020). The gut microbiome in coronary artery disease and heart failure: Current knowledge and future directions. EBioMedicine 52, 102649. 10.1016/j.ebiom.2020.102649 32062353 PMC7016372

[B47] Wallez Y. Cand F. Cruzalegui F. Wernstedt C. Souchelnytskyi S. Vilgrain I. (2007). Src kinase phosphorylates vascular endothelial-cadherin in response to vascular endothelial growth factor: Identification of tyrosine 685 as the unique target site. Oncogene 26 (7), 1067–1077. 10.1038/sj.onc.1209855 16909109

[B48] Wang Z. Klipfell E. Bennett B. J. Koeth R. Levison B. S. Dugar B. (2011). Gut flora metabolism of phosphatidylcholine promotes cardiovascular disease. Nature 472 (7341), 57–63. 10.1038/nature09922 21475195 PMC3086762

[B49] Wessel F. Winderlich M. Holm M. Frye M. Rivera-Galdos R. Vockel M. (2014). Leukocyte extravasation and vascular permeability are each controlled *in vivo* by different tyrosine residues of VE-cadherin. Nat. Immunol. 15 (3), 223–230. 10.1038/ni.2824 24487320

[B50] Wu X. W. Li G. Cheng X. B. Wang M. Wang L. L. Wang H. H. (2021). Association of angiotensin II type 1 receptor agonistic autoantibodies with outcomes in patients with acute aortic dissection. JAMA Netw. Open 4 (10), e2127587. 10.1001/jamanetworkopen.2021.27587 34596673 PMC8486983

[B51] Wu Z. Wang Z. Dai F. Liu H. Ren W. Chang J. (2016). Dephosphorylation of Y685-VE-cadherin involved in pulmonary microvascular endothelial barrier injury induced by angiotensin II. Mediat. Inflamm. 2016, 8696481. 10.1155/2016/8696481 PMC5227173 28119542

