# Supplementary material for: The short-chain fatty acid butyrate exerts a specific effect on VE-cadherin phosphorylation and alters the integrity of aortic endothelial cells
Source: Front Cell Dev Biol. 2023 Feb 8;11:1076250. doi: 10.3389/fcell.2023.1076250 (PMC9944439; doi:10.3389/fcell.2023.1076250)
Supplement: Supplementary file 1 [file Table1.DOCX]

Supplementary Material

# Supplementary Figures


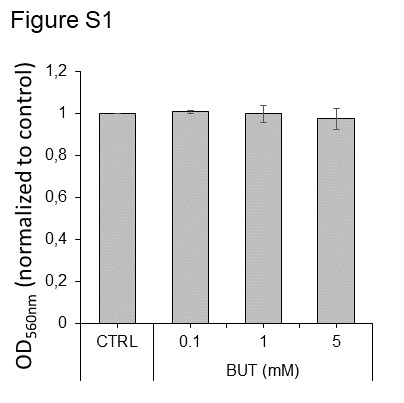


**Supplementary Figure S1: BUT exerts no cell toxic effects.** Influence of 1h of treatment of HAOEC with the given BUT concentrations on the viability and proliferation of HAOEC was measured by the MTT assay. The transversion of MTT reagent into its formazan complex was quantified measuring absorbance at OD_560nm_. Data represent mean ±SEM of n=4 experiments, performed in triplicate and normalized to control condition (no BUT treatment).


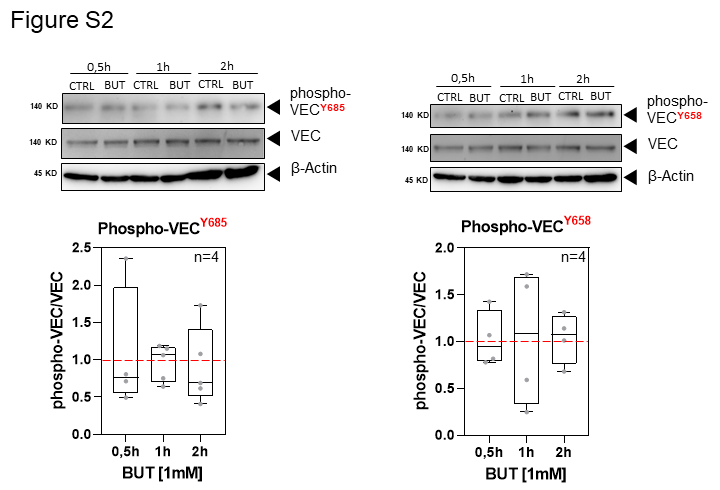


**Supplementary Figure S2: BUT did not significantly change phospho-VEC**^Y685, Y658^ **level in HAOEC.** Phospho-specific levels of VEC were analyzed and quantified as described in Fig. 2. Data display densiometric quantification of relative phospho-VEC and VEC signals normalized to the level in control cells (dashed red line) of n≥4 experiments. For better illustration, the identical data set for 1h treatment at 1mM from Fig. 2A (phospho-VEC^Y685^ and phospho-VEC^Y658^) is shown.

78

104


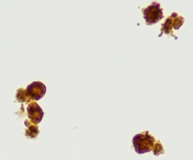

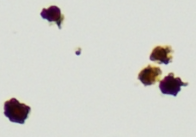


CTRLsiRNA

FFAR3siRNA

**Supplementary Figure S3: FFAR3 KD by siRNA**. Expression was FFAR3 expression upon transfection of HAOEC with control and specific siRNA by quantification of signal intensities of 8-bit images from IHC sections and FFAR3 staining (as described in Fig 3A). Quantification confirmed a decrease of 12.17% for FFAR3 expression. Total number of analyzed cells per conditions are displayed beneath the graph. Total numbers correspond to n=3 experiments.
